# Is Consciousness a Passive Recipient of the End-Product of Sophisticated Unconscious Computations?

**DOI:** 10.3389/fpsyg.2012.00413

**Published:** 2012-10-22

**Authors:** Pierre Perruchet, Bénédicte Poulin-Charronnat

**Affiliations:** ^1^Laboratoire d'Etude de l'Apprentissage et du Développement/Centre national de la recherche scientifique, Université de BourgogneDijon, France

The “hierarchical prediction machine” approach proposed by Clark shares with the standard computationalism the postulate of a powerful cognitive unconscious that “does the job.” All the possible options about the current percept are assumed to be computed and ordered as a function of their probability outside of any conscious experience, and, as we understand the approach, conscious thought is nothing else as the passive recipient of the ready-to-use end-result of the computation of multiple probability distributions.

The postulate of a smart cognitive unconscious is so deeply ingrained in our modern culture that it is taken for granted by most people. As a consequence, assuming the computation of multiple probability density distributions, keeping alive multiple options about the world, does not appear as less plausible than, say, imagining, in a Chomskyan framework, that the mind makes assumptions about the properties of the ambient language and tests hypotheses in order to set parameters of a Universal Grammar at their appropriate values. In both cases, consciousness appears as a supernumerary issue, something like the piece left over once the puzzle has been completed. In this regard, the place where the issue of consciousness is dealt with in the Clark's target paper, i.e., near of the end, is revealing.

Of course, there is no formal inconsistency in this view of the mind, as Clark argued. His subsequent claim that this view would *illuminate* the question of the content of our subjective experiences is more disputable. The reported example is the case of delusions and hallucinations in schizophrenia. Without undermining the importance of that issue, our feeling is that the questions emerging when the issue of the content of phenomenal experiences is put forward are primarily related to daily life and commonplace events. But our dissatisfaction with the place allocated to the issue of consciousness is much deeper. Our view is that, once the familiarity with the current cognitivist landscape has been put aside, the computational account of the mind, including the Bayesian approach, is singularly awkward. This account postulates an unconscious device endowed with extra powerful computational tools, whereas our conscious mental life would be condemned, for some mysterious reasons, to rely laboriously on far more limited capacities. Where is the mischievous sprite that prevents us from gaining deliberate access to the marvelous device that advocates of a Bayesian brain and other cognitive scientists imagine?

Clark is aware that postulating very sophisticated unconscious computations may be somewhat implausible, as revealed by the repeated mention that simpler mechanisms could act “as if” sophisticated operations were carried out. This is indeed an attractive solution, but the lack of concrete illustrations of how this could work, and the indeterminacy regarding how far these alternative mechanisms can go on the way of simplification, leaves the reader somewhat frustrated in this respect.

Another general model of the mind, presented 10 years ago in BBS (Perruchet and Vinter, 2002), proposed a much more radical departure with regard to the conventional cognitive framework. Paradoxically, this alternative approach found its origin in an earlier Clark's contribution to BBS (Clark and Thornton, 1997). In this earlier paper, Clark and Thornton showed how trading achieved representations against computational search could allow to easily solve problems that seemed intractable without some recoding of the incoming information. Perruchet and Vinter generalized this approach, and posited the formation of representations isomorphic to the world structure as the ultimate question that cognitive sciences have to address. Crucially, they postulated from the outset that all mental representations are conscious, on the basis of a twofold consideration. On the one hand, everyone knows that at least some mental events are conscious, because we have direct and personal evidence of their existence. Even those who argue that consciousness is epiphenomenal cannot reject this assessment. On the other hand, the existence of an unconscious mental life is a postulate or a presupposition, given that we have, by definition, no direct proof of an unconscious counterpart to our conscious mental life. It emerges from these two simple premises that, in striking contradiction with a widespread view among cognitive scientists, a framework relying exclusively on the representations and the mental operations we are aware of are more parsimonious than accounts postulating, in addition, a parallel cognitive apparatus. It is worth adding that exclusively relying on the conscious/attentional system brings out important functional constraints on the available explanatory tools, such as limited capacity, seriality of processing, and quick forgetting.

The approach outlined by Perruchet and Vinter (2002) meets the desired objective of accounting for conscious representations (roughly) isomorphic to the world structure thanks to the power of self-organizing principles. The progressive organization of the mind is assumed to emerge through elementary associative processes that take the conscious representations themselves as the stuff on which they operate, a general process that was summarized in the concept of self-organizing consciousness (SOC). The SOC model shares with the recent Clark's proposal a number of attractive features, such as bringing cognition, perception, and attention together within a unified framework. The SOC model is also based on the interplay of top-down and bottom-up influences: The nature of the processing primitives determines how the world is perceived and the nature of the world determines the transformation of the processing primitives, and so on, recursively, in line with a developmental principle initially described by Piaget's concepts of assimilation and accommodation. Finally, the SOC model also accounts for the human high responsiveness to the statistical structure of our environment. Admittedly, this model is less developed and polished than the hierarchical prediction machine described by Clark, especially with regard to its possible implementation within the neural machinery. However, as an outline of a further full-blown model, the Perruchet and Vinter's proposal has, in our view, the crucial advantage of accounting for mental life without any call for unconscious computations, making it closer to the “neats” than to the “scruffies” in a more obvious way than Clark's proposal.
